# Investigating urban soundscapes of the COVID-19 lockdown: A predictive soundscape modeling approacha)

**DOI:** 10.1121/10.0008928

**Published:** 2021-12-28

**Authors:** Andrew Mitchell, Tin Oberman, Francesco Aletta, Magdalena Kachlicka, Matteo Lionello, Mercede Erfanian, Jian Kang

**Affiliations:** Institute for Environmental Design and Engineering, The Bartlett, University College London, London, United Kingdom

## Abstract

The unprecedented lockdowns resulting from COVID-19 in spring 2020 triggered changes in human activities in public spaces. A predictive modeling approach was developed to characterize the changes in the perception of the sound environment when people could not be surveyed. Building on a database of soundscape questionnaires (*N* = 1,136) and binaural recordings (*N* = 687) collected in 13 locations across London and Venice during 2019, new recordings (*N* = 571) were made in the same locations during the 2020 lockdowns. Using these 30-s-long recordings, linear multilevel models were developed to predict the soundscape pleasantness ( $R^{2}=0.85$) and eventfulness ( $R^{2}=0.715$) during the lockdown and compare the changes for each location. The performance was above average for comparable models. An online listening study also investigated the change in the sound sources within the spaces. Results indicate (1) human sounds were less dominant and natural sounds more dominant across all locations; (2) contextual information is important for predicting pleasantness but not for eventfulness; (3) perception shifted toward less eventful soundscapes and to more pleasant soundscapes for previously traffic-dominated locations but not for human- and natural-dominated locations. This study demonstrates the usefulness of predictive modeling and the importance of considering contextual information when discussing the impact of sound level reductions on the soundscape.

## I. INTRODUCTION

The global emergency caused by the COVID-19 pandemic in early 2020 required national lockdown measures across the world, primarily targeting human activity. In the United Kingdom, construction and transport were allowed to continue, but a decrease in activity was observed (Hadjidemetriou *et al.*, 2020). In other countries, such as Italy, the restrictions were more severe and even included limiting people's movement to a certain radius from their place of residence (Ren, 2020). The explorations in environmental acoustics of lockdown conditions across the world have revealed various degrees of impact on the acoustic environment with researchers reporting reductions in noise levels affecting the population at the scale of urban agglomerations such as Ruhr Area in Germany (Hornberg *et al.*, 2021) and conurbations in the south of France (Munoz *et al.*, 2020). Impacts have also been reported at a scale of a multimillion city such as Madrid (Asensio *et al.*, 2020b) or Barcelona (Bonet-Solà *et al.*, 2021), as well as at a more local, city-center or even public space-scale in cities such as Stockholm (Rumpler *et al.*, 2021), London (Aletta *et al.*, 2020), Girona (Alsina-Pagès *et al.*, 2021), or Granada (Vida Manzano *et al.*, 2021). In general, these studies have demonstrated a decrease in urban noise levels and indicated a difference in the amount that the level decreased, depending on the type of space investigated (e.g., parks, urban squares, etc.) and the type of human activity characteristic for the space, with higher reductions in places typically associated with human sounds and activities such as shopping and tourism.

Those studies were mostly focused around the *L_A_*_eq_, as well as a standardization approach to reporting subsequent changes in the soundscape, proposed by Asensio *et al.* (2020a). They were not able to reveal the perceptual impact of such conditions in public spaces because of (1) the lack of subjective data for the exact or comparable locations in previous years; and (2) the lack of participants present in public spaces during the lockdown, hence, the inability to collect soundscape data *in situ*. Munoz *et al.* (2020) combined noise measurements with an online questionnaire deployed to residents, some of which were residing in the areas covered by the noise monitoring network available. The participants were asked to recall how their lockdown area sounded before and during the first lockdown in 2020 and describe the perceived change. They observed a consistent reduction in levels, followed by the perceived reduction of transport sounds (air and road) and an increase in natural sounds, whereas the resulting environment was described as pleasant, calm, and peaceful. By combining field recordings and focus groups, Sakagami (2020) and Lenzi *et al.* (2021) observed changes in the sound source composition and the affective quality of the soundscape in a residential area in Kobe, Japan and a public space in Getxa, Spain, respectively, during the different stages of the lockdown period. Following the easing of the lockdown measures, a decrease in animal and traffic sounds was observed in Kobe, whereas an increase in eventfulness, loudness, and presence of human sound sources, followed by a decrease in pleasantness, was shown in Getxa.

Aletta *et al.* (2020) explored the impacts of the COVID-19 lockdowns on the acoustic environment in London, in particular, through many short-term (30 s) binaural recordings. This study revealed that average reductions in the various locations considered ranged from 10.7 (*L_A_*_eq_) to 1.2 dB with an overall average reduction of 5.4 dB. This metric-reporting focused approach left the following research questions unanswered: how would people have perceived these spaces as a result of this change in the acoustic environment? (RQ1) and would these sound level reductions result in improvements to the soundscape of the spaces (RQ2)? The first research question (RQ1), addressing the perceptual effect of the change in the urban soundscape induced by the lockdowns, can be further broken down into the following questions: how was the sound source composition influenced by the change?; how would the affective response to the acoustic environment in the lockdowns change?; and could this demonstrate the effect of human activities on the perception of an acoustic environment in general?

These questions arise out of the soundscape approach, which is characterized by prioritizing the perceptual effect of an acoustic environment by taking into account the interaction of the sound sources, context, and the person perceiving it (ISO, 2014; Truax, 1999), bringing together objective and subjective factors. The soundscape approach to noise mitigation and management is being recognized as a response to the arising environmental requirements on noise pollution and sustainability such as the regulation of quiet areas in Europe (European Environment Agency, 2020; Kang and Aletta, 2018; Radicchi *et al.*, 2021). This has been further formalized in ISO/TS (2018) via the adoption of the circumplex model of soundscape (Axelsson *et al.*, 2010), in which the perception of a soundscape can be described in terms of its pleasantness and eventfulness, as one of the standard methods of soundscape assessment.

Soundscape research is, therefore, traditionally rooted in environmental acoustics and environmental psychology, typically dealing with outdoor spaces (Torresin *et al.*, 2020) and urban open spaces, where parks and squares are often used as case study sites (Kang, 2007). A soundscape assessment typically requires people to be surveyed, but the presence of people at a location influences the assessment (Aletta and Kang, 2018) and “quiet places” usually require low numbers of users to remain quiet, which limits the possibility of an assessment. Even in a crowded public space, soundscape surveys are demanding as they require significant resources to perform at scale, limiting their widespread application (Mitchell *et al.*, 2020). Therefore, a need for a predictive model arises to overcome this limitation and improve the implementation of the soundscape approach into everyday planning and management practices.

According to a recent review of predictive soundscape models from Lionello *et al.* (2020), the degree of employing auditory and nonauditory factors in soundscape prediction varies with some studies relying on contextual, personal/demographic (Erfanian *et al.*, 2021; Tarlao *et al.*, 2021), or social media (Aiello *et al.*, 2016) data entirely to predict and generate the soundscape features. Some methods also incorporate perceptually derived features, such as subjective sound level and visual pleasantness, as predictors (Lionello *et al.*, 2020). In general, these methods, which incorporate perceptually derived inputs, achieve better accuracy rates than those which do not; however, this perception information must also be obtained from people via a survey and, therefore, are unsuitable for predictive modeling, where surveys are not possible. For example, Ricciardi *et al.* (2015) proposed two models based on data collected from a smartphone application to predict urban sound quality indicators based on linear regressions. The first model, which incorporated perceptually derived input features (visual quality and familiarity), achieved an *R*^2^ of 0.72, whereas a second model without these features achieved an *R*^2^ of 0.58. This indicates the necessity for considering and accounting for the influence which contextual factors in a space have on the relationship between the sound environment itself and the listener's perception of it (i.e., the soundscape) while also highlighting the challenges associated with a predictive model, which depends only on measurable features.

Therefore, a third research question arises: what are the key features needed for a soundscape prediction model based on comprehensive acoustic on-site measurements to be used for assessing locations with low social presence or in situations where conducting surveys is impractical (RQ3)?

## II. MATERIALS AND METHODS

This study was conducted via initial on-site data collection campaigns in Central London and Venice in 2019 before the outbreak of COVID-19 as part of the Soundscape Indices (SSID) project (Mitchell *et al.*, 2020) and in 2020 during the strictest part of the lockdowns (Aletta *et al.*, 2020), including objective acoustic data (2019 and 2020) and subjective responses (2019 only). The full *in situ* dataset, as described in this section, has been made publicly available as “The International Soundscape Database (V0.2.1)” on Zenodo^1^ (Mitchell *et al.*, 2021).

Using the 2019 and 2020 binaural recordings, an online listening experiment was conducted to provide an understanding about the change in the sound source composition. The 2019 on-site questionnaire data were used to define the dominant sound source at each location as a starting point for interpreting the soundscape change. A predictive model was developed to reveal the change in the perceived pleasantness and eventfulness using the objective acoustic data and location to predict the subjective responses. Although the initial (2019) dataset contains additional locations (specifically, in Spain, the Netherlands, and China), due to the nature of this study as a reaction to the strict movement and activity restrictions, the sites which could be included in the lockdown (2020) measurement campaigns were limited to the locations where the staff and equipment had access and recordings could be undertaken during the spring of 2020.

The sites were selected to provide a mixture of sizes and uses, varying in typology and ranging from paved squares to small and large parks to waterside spaces across both cities. Throughout the text, they are indexed via a “LocationID” based on the location's name (e.g., CamdenTown, SanMarco), and a more in-depth overview of each is given in the supplementary material.^2^ London is taken as an example of a large, typically noisy city and the Venice sample provides a unique look at spaces with typically very high human activity levels and no road traffic activity. In particular, the 2019 Venice surveys were taken to coincide with the yearly Carnevale festival to capture its distinct soundscape.

The ISO/TS (2018) series was consulted for reporting on the soundscape data. A detailed description of the 2019 survey campaigns is featured throughout the paper and in the public database.^1^ This study was approved by the departmental University College London (UCL) Institute for Environmental Design and Engineering (IEDE) Ethics Committee on 17 July 2018 for the on-site data collection and 2 June 2020 for the online listening experiment and is conducted in adherence to the ethical requirements of the Declaration of Helsinki (World Medical Association, 2013).

### A. On-site data: Questionnaires, binaural measurements, and recordings

The initial on-site data collection featured questionnaire data collected from the general public and acoustic measurements, conducted across 13 urban locations (in London *N* = 11, in Venice *N* = 2) between 28 February and 21 June 2019 with additional sessions in July and October 2019. Although the total survey period in 2019 extended over several seasons, the surveys at any individual location did not extend over seasons with different occupancy patterns. A total of 1318 questionnaire responses were collected from the general population across the measurement points during 1–3-h-long campaigns in both cities in 2019, accompanied by 693 approximately 30-s-long 24-bit 44.1 kHz binaural recordings. After data cleaning, each of the 13 locations was characterized by between 14 and 80 recordings and between 24 and 147 questionnaire responses. The mean age of the participants was 33.8 years old with a standard deviation of 14.57 (45% male, 53.8% female, 0.4% nonconforming, 0.9% prefer-not-to-say).

Although recent results from both Tarlao *et al.* (2021) and Erfanian *et al.* (2021) indicate the important influence of personal and demographic factors—in particular, age and gender—on the soundscape perception, these factors were not included as potential features in the modeling process. Given the nature of this study as addressing a scenario when people could not be surveyed, no additional demographic information is available in the lockdown case to be fed into the model and is, therefore, not useful to include for the development and application of this specific predictive model. This information is reported throughout the study simply to provide further context to the data collection.

The subsequent measurement campaign in 2020 mimicked the binaural recording strategy applied in the initial campaign and was performed between 6 and 25 April 2020 in both cities, this time excluding the questionnaire. An additional 571 binaural recordings were collected on-site in 2020.

#### 1. Data collection

The 2019 data collection was performed across all of the locations using the protocol based on Method A of ISO/TS (2018) as described in Aletta *et al.* (2020) and Mitchell *et al.* (2020), collected either via handheld tablets or paper copies of the questionnaire. The full questionnaire and data collection procedure are given in Mitchell *et al.* (2020); however, the key parts used for this study are those addressing the sound source dominance and perceived affective quality (PAQ).

The participants are first asked to rate the perceived dominance of several sound sources as assessed via a five-point Likert scale, coded from one (not at all) to five (dominates completely). The sound sources are split into four categories, traffic noise, other noise, human sounds, and natural sounds, and each is rated separately. Next are the eight PAQs, which make up the circumplex model of soundscape (Axelsson *et al.*, 2010): pleasant, chaotic, vibrant, uneventful, calm, annoying, eventful, and monotonous. These are assessed on a five-point Likert scale from one (strongly disagree) to five (strongly agree). To simplify the results and allow for modeling the responses as continuous values, the eight PAQs undergo a trigonometric projection to reduce them onto the two primary dimensions of pleasant and eventful, according to the procedure outlined in Part 3 of the International Organization for Standardization (ISO) 12913 series (ISO/TS, 2019). To distinguish the projected values from the Likert-scale PAQ responses, the projected values will be referred to as ISOPleasant and ISOEventful and can be considered to form an *x*-*y* coordinate point (*x* = ISOPleasant, *y* = ISOEventful) as explained in detail in Lionello *et al.* (2021).

The calibrated binaural device SQobold with BHS II by Head Acoustics (GmbH, Herzogenrath, Germany) was used in both campaigns at all of the locations by various operators to capture the acoustic data as mentioned in the Acknowledgments. Following the established on-site protocol (Mitchell *et al.*, 2020), when participants were stopped in a group and filled in their responses simultaneously, a single binaural recording was used to capture their experience as a group. The purpose behind this sampling strategy was to obtain data from the perspective of a typical user, corresponding to a range of individual experiences available within an urban open space. These recordings are indexed by a “GroupID” such that the recording for each group is matched up to each of the corresponding respondents and their individual survey responses.

#### 2. Data cleaning

The cleaning of the samples was conducted using the ArtemiS SUITE 11 (HEAD Acoustics GmbH, Herzogenrath, Germany). The researcher discarded or cropped whole recordings or its parts affected by wind gusts or containing noises and speech generated by the recording operator by accident or for the purpose of explaining the questionnaire to a participant. This resulted in 1258 binaural recordings, which were then processed further as described in Sec. II A 3. The psychoacoustic analyses are shown in the publicly available database.^1^

To maintain the data quality and exclude the cases in which the respondents either clearly did not understand the PAQ adjectives or intentionally misrepresented their answers, surveys for which the same response was given for every PAQ (e.g., “strongly agree” to all eight attributes) were excluded prior to calculating the International Organization for Standardization (ISO) projected values. This is justified as no reasonable respondent who understood the questions would answer that they “strongly agree” that a soundscape is pleasant and annoying, calm, and chaotic, etc. The cases in which the respondents answered “neutral” to all of the PAQs are not excluded in this way, as a neutral response to all attributes is not necessarily contradictory. In addition, surveys were discarded as incomplete if more than 50% of the PAQ and sound source questions were not completed.

The site characterization per ISO/TS (2018) is available in the supplementary material^2^ and public database,^1^ featuring the address, overall psychoacoustic characteristics of the location, typical use of each location, and pictures taken during the survey sessions.

#### 3. Psychoacoustic analyses

The binaural recordings were analyzed in ArtemiS SUITE 11 (HEAD Acoustics GmbH, Herzogenrath, Germany) to calculate the suite of 11 acoustic and psychoacoustic features given in Table I to be used as the initial predictors. The (psycho)acoustic predictors investigated were selected to describe the many aspects of the recorded sound, in particular, the goal was to move beyond a focus on the sound level, which currently dominates the existing literature on the acoustic effects of lockdowns noted in Sec. I. In all, they are expected to reflect the sound level (*L_A_*_eq_), perceived sound level (loudness), spectral content (sharpness, $L_{C\text{eq}}-L_{A\text{eq}}$, tonality), temporal character, or predictability (impulsiveness, fluctuation strength, relative approach), and overall annoyance (psychoacoustic annoyance). These metrics have been proposed as indicators to predict the perceptual constructs of the soundscape (Aletta *et al.*, 2016; Aletta *et al.*, 2017) and have shown promise when combined together to form a more comprehensive model applied to real-world sounds (Orga *et al.*, 2021). The maximum value from the left and right channels of the binaural recording are used, as suggested in ISO/TS (2019).

**TABLE I.** The psychoacoustic features considered for inclusion in the predictive models. All of the metrics are calculated for the full length of the recording (∼30 s). As recommended by ISO (2017) and ISO/TS (2018), the fifth percentile of loudness is used rather than the average.

Feature	Symbol	Unit	Calculation method
Loudness (fifth percentile)	*N*_5_	sones	ISO (2017)
Sharpness	*S*	acum	ISO (2017)
Roughness	*R*	asper	ECMA (2020)
Impulsiveness	*I*	iu	ECMA (2020)
Fluctuation strength	FS	vacil	ECMA (2020)
Tonality	*T*	tuHMS	Sottek (2016)
Psychoacoustic annoyance	PA	—	Zwicker and Fastl (2007)
*L_A_*_eq_	*L_A_*_eq_	dB	IEC (2013)
$L_{A10}-L_{A90}$	$L_{A10}-L_{A90}$	dB	ISO (2016)
$L_{\mathrm{Ceq}}-L_{\mathrm{Aeq}}$	$L_{C\text{eq}}-L_{A\text{eq}}$	dB	ISO (2016)
Relative approach	RA	cPA	Sottek and Genuit (2005)

Table II shows the Pearson correlation coefficient between each of the candidate acoustic features and the outcome pleasantness and eventfulness. As all of the variables considered are continuous, and the eventual model is linear, the Pearson coefficient is chosen as a measure of the strength of the linear relationship between two continuous variables. For ISOPleasant (ISOPl), we can, perhaps, see three tiers of correlations: the more highly correlated tier (|r|>0.28) consists of the relative approach (RA), *L_A_*_eq_, roughness (*R*), loudness (*N*_5_), and psychoacoustic annoyance (PA); the low correlation tier consists of $L_{A10}-L_{A90}$, tonality (*T*), and fluctuation strength (FS); whereas $L_{C\text{eq}}-L_{A\text{eq}}$, impulsiveness (*I*), and sharpness (*S*) show no correlation. For ISOEventful (ISOEv), these tiers are RA, *L_A_*_eq_, *T*, *R*, and *N*_5_ comprise the most correlated tier (|r|>0.30); $L_{C\text{eq}}-L_{A\text{eq}}$, $L_{A10}-L_{A90}$, FS, and PA show low correlations; *I* and *S* show no correlation.

**TABLE II.** The Pearson correlation coefficients between the candidate acoustic features and ISOPleasant and ISOEventful across all 13 locations. Only the statistically significant (*p* < 0.01) coefficients are shown.

Parameter	ISOPl	ISOEv	PA	*N*_5_	*S*	*R*	*I*	FS	*T*	*L_A_*_eq_	$L_{A10}-L_{A90}$	$L_{C\text{eq}}-L_{A\text{eq}}$
ISOPleasant												
ISOEventful	−0.24											
PA	−0.28	0.24										
*N*_5_	−0.37	0.33	0.94									
*S*			0.71	0.56								
*R*	−0.36	0.32	0.63	0.74	0.11							
*I*			−0.10		−0.37	0.24						
FS	−0.11	0.14	0.37	0.43		0.46	0.55					
*T*	−0.21	0.30	0.58	0.63	0.12	0.54	0.16	0.52				
*L_A_*_eq_	−0.34	0.37	0.84	0.93	0.56	0.72	−0.09	0.37	0.57			
$L_{A10}-L_{A90}$	−0.18	0.15	0.21	0.33	−0.20	0.31	0.36	0.44	0.40	0.23		
$L_{C\text{eq}}-L_{A\text{eq}}$		−0.20	−0.49	−0.49	−0.54	−0.31		−0.27	−0.28	−0.61	−0.22	
RA	−0.34	0.31	0.60	0.74	0.18	0.71	0.31	0.63	0.58	0.73	0.23	−0.14

Among the intercorrelations for the psychoacoustic metrics considered for inclusion as input features, we can see several very highly correlated features (i.e., >0.9). As expected, PA, *L_A_*_eq_, and *N*_5_ are highly correlated, meaning that careful consideration is paid to these features to ensure that they do not contribute to multicollinearity in the final model.

### B. Modeling

Two linear multilevel models (MLM) were computed to predict (1) ISOPleasant and (2) ISOEventful. These models are trained on the 2019 data only, and then applied to the acoustic data collected during the 2020 lockdowns, the results of which are reported in Sec. III. The inherent grouped structure of the SSID database necessitates a modeling and analysis approach which considers the differing relationships between the objective acoustic features and the soundscape's PAQ ratings across the various locations and contexts. The individual-level of the models is made up of the acoustic features calculated from the binaural recordings made during each respondent's survey period, whereas the group-level includes the categorical LocationID variable, indicating the location in which the survey was taken, acting as a nonauditory contextual factor.

A separate backward-step feature selection was performed for each of the outcome models to identify the minimal feature set to be used for predicting each outcome. In this feature selection process, an initial model containing all of the candidate features was fit. Each feature was then removed from the model one at a time, then the best-performing model is selected, and the procedure continues stepwise until no improvement is seen by removing more features. This process is performed first on the location-level features (including the potential to remove all features, including LocationID, resulting in a “flat” or standard multivariate linear regression model), and then on the individual-level features. The performance criterion used for this process was the Akaike information criterion (AIC; Akaike, 1974). To check for multicollinearity among the selected features, the variance inflation factor (VIF) was calculated and a threshold of VIF < 5 was set. Any features which remained after the backward stepwise selection and exceeded this threshold were investigated and removed if they were highly collinear with the other features.

All of the input features are numeric values in the units described above. Before conducting the feature selection, the input features are *z*-scaled to enable a proper comparison of their effect sizes. After the feature selection, the scaled coefficients are used in the text when reporting the final fitted models to facilitate the discussion and comparison between the features. The unscaled model coefficients are reported in Appendix B to enable the models to be applied to new data. To properly assess the predictive performance of the model, an 80/20 train-test split with a balanced shuffle across LocationIDs was used. The *z*-scaling and feature selection were performed on the training set only to prevent data leakage. To score the performance of the model on the training and testing sets, we use the mean absolute error (MAE), which is in the scale of the response feature—for ISOPleasant, this means our response can range from -1 to +1. However, because the end-goal of the model is to predict the soundscape assessment of the location as a whole, rather than the individual responses, we also assess the performance of the model in predicting the average response in each location. To do this, the mean response value for each location is calculated, and the *R*^2^ accuracy across LocationIDs is reported for the training and testing sets.

The model fitting and feature selection was performed using the “step” function from lmerTest (v3.1.3; Kuznetsova *et al.*, 2017) in *R* statistical software (v4.0.3; *R* Core Team, 2020). The summaries and plots were created using the sjPlot package (v2.8.6; Lüdecke, 2021) and seaborn (v0.11.1; Waskom, 2021).

### C. Online survey

An online listening test was conducted using the Gorilla Experiment Builder^3^ (Anwyl-Irvine *et al.*, 2020). The participants were exposed to a random selection of 78 binaural recordings (39 from 2019 and 39 from 2020, 6 recordings per location). Each participant had the option to evaluate either 1 or 2 sets of 6 recordings randomly assigned between 13 stimuli sets. mp3 files, converted at 256 kBps, were used due to the requirements of the Gorilla platform.

No visual stimuli were used in the experiment. The experiment consisted of (1) an initial exercise to enhance the chances of participants complying to the instructions and wearing headphones; (2) a training set using two randomly chosen binaural recordings (then not used in the main task) from the dataset; (3) a soundscape characterization questionnaire starting with an open-ended question about the perceived sound sources and featuring the same questions as the one used *in situ*, looking into the PAQ of the soundscape and the perceived sound source dominance of the following four types: traffic noise, other noise, human sounds, and natural sounds; (4) a questionnaire on the basic demographic factors. The questionnaire used in part (3) of the online experiment is reported in Appendix A.

Keeping in mind the remote nature of the study and ensuring a minimum level of robustness for reliable sound source recognition, an initial exercise was performed, consisting of a headphone screening test (Woods *et al.*, 2017) and a headphone reproduction level adjustment test (Gontier *et al.*, 2019). The level adjustment was performed using an 11-s-long pink noise sample matched to the lowest and highest $L_{A90}$ values from the experimental set. The participants were asked to adjust their listening level to clearly hear the quieter sample while keeping the level low enough that they do not find the louder sample disturbing. The headphones screening test followed, featuring a stereo signal of a 1-s-long 100 Hz sine tone, generated with Izotope RX 6 application (Izotope, Inc, Camgridge, MA), played at a 3 dB difference, where one of the equally loud pairs had its phase inverted. A 100 Hz sine was used because the pilot tests revealed that the 200 Hz sine tone proposed by Woods *et al.* (2017) created a higher uncertainty, varying across different laptop models, and would likely contribute to the chances of a participant fooling the test. It was expected that the participants using speakers would not be able to hear the sine wave or would be fooled by the inverted phase effect and, therefore, not able to pass the trials unless they were indeed using headphones. The participants needed to recognize the quietest of the three samples in a trial of six attempts. Only participants correctly answering five or more out of six trials were allowed to proceed with the experiment. The participants were asked not to change their audio output settings during the remainder of the experiment. (This was introduced to ensure that a participant is using a headphones playback system, which allows a listener to clearly recognize a 3 dB difference at 100 Hz as a proxy for sufficient audio quality playback.)

However, after the initial data collection, questions were raised as to how the playback loudness impacts the ecological validity as it relates to the PAQ of the soundscape. Given this concern, the PAQ responses from the online surveys were not included in further data analysis. Sound source identification is not considered to suffer the same validity concerns as this is not directly dependent on the absolute playback level and requires only that the participant can clearly hear what is presented. The purpose of the calibration procedure described above was to ensure that the participant could clearly hear the softest samples used.

The online questionnaire data were collected between 9 June and 9 August 2020. Within the Gorilla Experiment Builder, a total of 250 attempts to complete the experiment were recorded, where 165 participants were excluded either on the basis of not passing the headphones screening (*N* = 79) or not completing the experiment, usually before engaging into the screening (*N* = 83). Out of a total of 88 participants who completed the test, 2 participants were excluded as outliers as they provided uniform answers across all of the questions and commented on not being able to properly hear the stimuli despite their successful completion of the training tests. The participants of the online experiment were of the mean age of 32.42 years old and were 45.1% male and 54.9% female.

**FIG. 1.**
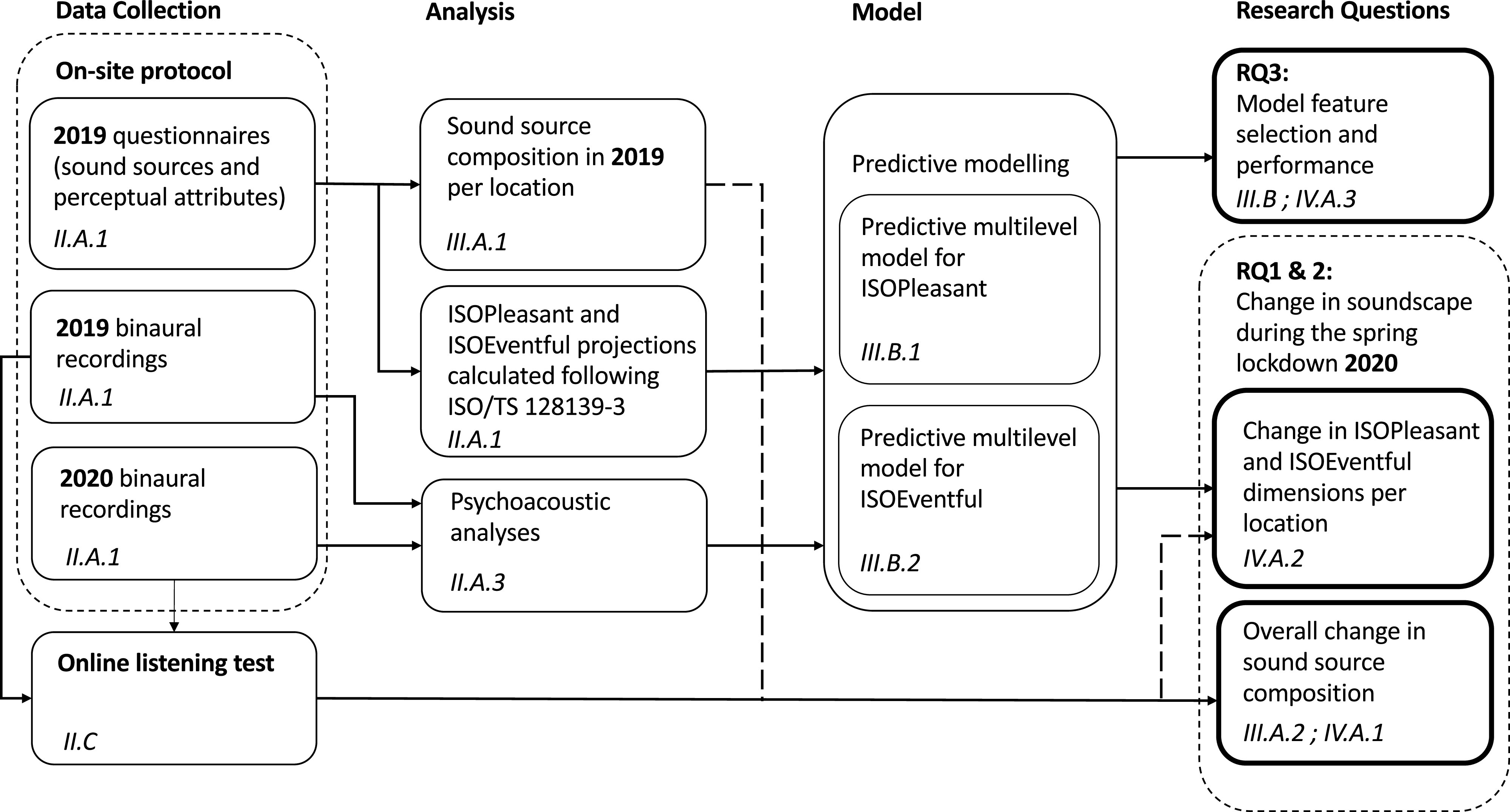
The study flow chart indicating the data collection, analysis, modeling, and discussion throughout the study. The subsections in the text to which each box refers are indicated in italic.

Figure 1 illustrates and summarizes the framework and sections described above.

## III. RESULTS

The results of the on-site surveys, online experiment, and the model development are reported here. They are reported following the structure of the ISO/TS 12913 series, revealing the perceived sound source dominance, key perceptual attributes (ISOPleasant and ISOEventful), and the lockdown-related changes.

### A. Perceived sound source dominance

#### 1. 2019 sound source composition per location

The questionnaire data were collected in English, Italian, and Spanish in London and Venice. The respective questionnaires can be found in the supplementary material2 and Mitchell *et al.* (2020). The data presented here were aggregated per the LocationID.

**FIG. 2.**
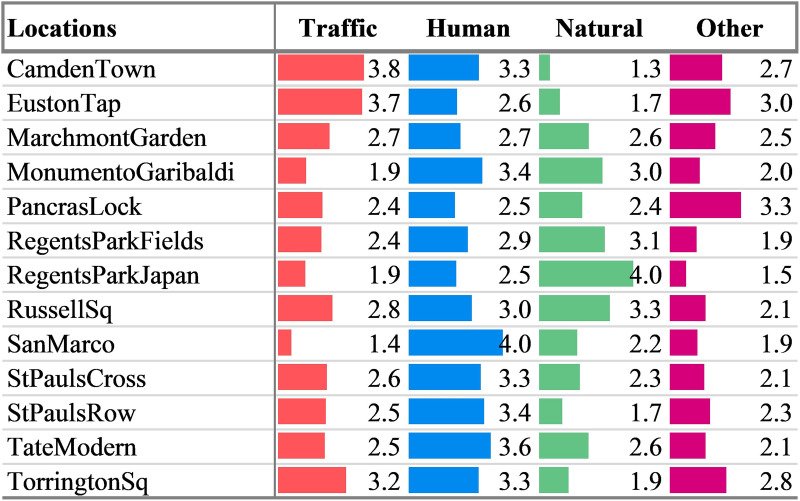
(Color online) The mean response per location ID for the perceived dominance of the sound source types for the 2019 on-site campaign. The values represent the mean response of all of the participants in each location to the question “To what extent do you presently hear the following four types of sounds?”. The response values range from (1) not at all to (5) dominates completely.

According to the highest scored mean value of the dominant sound source type, as shown in Fig. 2, the locations can be grouped into natural sounds dominated (RegentsParkJapan, RegentsParkFields, RussellSq), human sounds dominated (SanMarco, TateModern, StPaulsRow, StPaulsCross, MonumentoGaribaldi), and noise (traffic and other noise) sounds dominated (CamdenTown, EustonTap, TorringtonSq, PancrasLock). Traffic noise and other noise have been combined here and for the rest of the discussion, as these responses are highly correlated within this dataset and it is not helpful to consider them separately for this analysis. This follows the alternative sound source labels given in Fig. C.3 of ISO/TS (2018), which combines traffic and other noise. Finally, MarchmontGarden is unique in that all of the sound source types are assessed as being nearly equally present with only 0.2 separating the least present (other noise, 2.5) and the most present (traffic noise, 2.7).

#### 2. Overall change in the perceived sound source dominance during lockdown

**FIG. 3.**
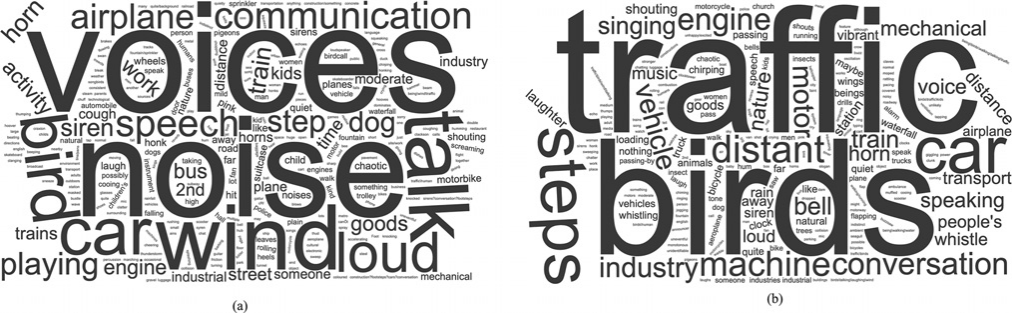
A graphic illustrating the frequency of occurrence of the sound sources reported by the participants of the online study across all locations, shown for recordings from 2019 (left) and 2020 (right).

1803 words describing the sound sources present in the 2019 recordings and 1395 words related to the 2020 recordings were input by participants in response to the open-ended question Q1 (see Appendix A). The frequency of occurrence, generated using the WordClouds web application (Wordclouds.com, Zygomatic, Vianen, NL), is shown in Fig. 3 for the 2019 and the 2020 sets. The most frequent words from the 2019 and 2020 groups are noise, car/traffic, bird/birds, talk/voice, and (foot)steps.

The results from the listening tests deployed online were analyzed using the SPSS Statistics v.25 (IBM United Kingdom Limited, Portsmouth, UK; see Table III). Levene's test for equality of variances resulted in highly statistically significant values for all four sound sources investigated (less than 0.001). Therefore, a Mann-Whitney *U*-test test was used as a nonparametric equivalent to the *T*-test to investigate the change in the perceived dominance of the four sound source types (McKnight and Najab, 2010). The results for human sounds indicated that the perceived dominance was greater for the 2019 sample (*M* = 3.82) than for the 2020 sample (*M* = 2.62), *U* = 41 656, *p* < 0.001. The results for natural sounds indicated the perceived dominance increased from 2019 (*M* = 2.00) to 2020 (*M* = 2.54), *U* = 63 797, *p* < 0.001. However, the differences for the noise sources (traffic and other) were not statistically significant. The result of these changes is that although human sounds were the clearly dominant source across the whole dataset in 2019, in 2020, the sound sources are, on average, much more evenly balanced. No single sound source category was identified as frequent across the 2020 dataset.

**TABLE III.** The mean values and standard deviations for the perceived dominance of sound sources (rated from one to five), assessed via an online survey.

Sound source type	Campaign	*N*	Mean	Standard deviation	Standard error mean
Traffic	2019	422	2.51	1.369	0.067
	2020	383	2.56	1.525	0.078
Other	2019	422	2.00	1.182	0.058
	2020	382	2.23	1.333	0.068
Human	2019	423	3.82	1.143	0.056
	2020	382	2.62	1.346	0.069
Natural	2019	424	2.00	1.307	0.063
	2020	380	2.54	1.441	0.074

### B. Model selection, performance, and application

#### 1. ISOPleasant model selected

Following the feature selection, the ISOPleasant model (given in Table IV) has *N*_5_ as the fixed effect with a scaled coefficient of -0.06, and *L_A_*_eq_, $L_{A10}-L_{A90}$, and $L_{C\text{eq}}-L_{A\text{eq}}$ as coefficients which vary depending on the LocationID. The training and testing MAE are very similar, indicating that the model is neither over- nor underfitting to the training data (MAE_train_ = 0.258; MAE_test_ = 0.259). The model performs very well at predicting the average soundscape assessment of the locations ( $R_{\text{train}}^{2}=0.998$; $R_{\text{test}}^{2}=0.85$).

**TABLE IV.** The scaled linear regression models of ISOPleasant and ISOEventful for 13 locations in London and Venice. ISOPleasant model structure, random slope, random intercept multilevel model (MLM); ISOEventful model structure, multivariate linear regression. Statistically significant *p*-values are highlighted in bold.

	ISOPleasant			ISOEventful		
Predictors	Estimates	Confidence Interval (CI)	*p*	Estimates	CI	*p*
(Intercept)	0.24	0.15–0.33	**<0.001**	0.14	0.12–0.16	**<0.001**
*N*_5_	−0.06	−0.10–0.02	**<0.001**			
*S*				−0.08	−0.11–0.06	**<0.001**
FS				−0.02	−0.05–0.00	**0.033**
*T*				0.04	0.01–0.07	**0.002**
*L _A_*_eq_				0.14	0.11–0.17	**<0.001**
$L_{C\text{eq}}-L_{A\text{eq}}$				−0.03	−0.05–0.00	0.052
Random effects						
$\sigma^{2}$	0.11					
*τ*_00_	$0.03_{\text{LocationID}}$					
*τ*_11_	$0.02_{\text{LocationID}.L_{A\text{eq}}}$					
	$0.00_{\text{LocationID}.L_{A10}-L_{A90}}$					
	$0.00_{\text{LocationID}.L_{C\text{eq}}-L_{A\text{eq}}}$					
ICC	0.90					
*N*	$13_{\text{LocationID}}$					
Observations	914			914		
MAE train, test	0.258	0.259		0.233	0.231	

**FIG. 4.**
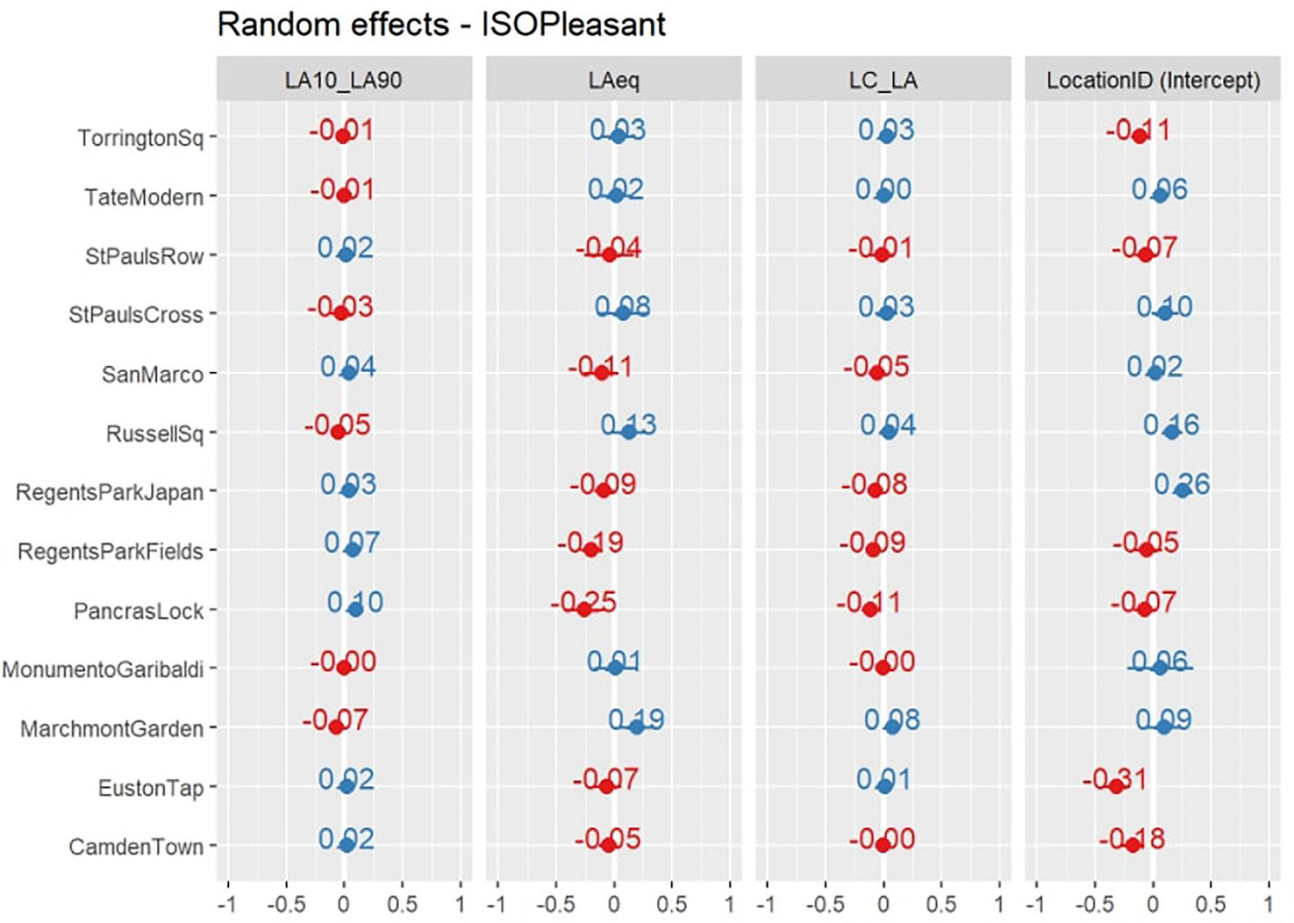
(Color online) The location-level scaled coefficients for the ISOPleasant model.

The high intraclass correlation (ICC = 0.90) demonstrates that the location-level effects are highly important in predicting the pleasantness dimension. Within this random-intercept random-slope model structure, these effects include the specific context of the location (i.e., the LocationID factor) and also the *L_A_*_eq_, $L_{A10}-L_{A90}$, and $L_{C\text{eq}}-L_{A\text{eq}}$ features, whose effects vary across the locations. These slopes are given in Fig. 4. This point highlights the need to consider how the context of a location will influence the relationship between the acoustic features and the perceived pleasantness.

#### 2. ISOEventful model selected

Through the group-level feature selection, all of the group-level coefficients were removed, including the LocationID factor itself. Therefore, the final ISOEventful model is a flat multivariate linear regression model rather than a MLM. The ISOEventful model is a linear combination of *S*, FS, *T*, *L_A_*_eq_, and $L_{C\text{eq}}-L_{A\text{eq}}$. The training and testing MAEs are very similar, indicating that the model is not overfit to the training data (MAE_train_ = 0.233; MAE_test_ = 0.231). The model performs slightly worse than the ISOPleasant model at predicting the mean location responses but still performs well ( $R_{\text{train}}^{2}=0.873$; $R_{\text{test}}^{2}=0.715$).

#### 3. Application to the lockdown data

**FIG. 5.**
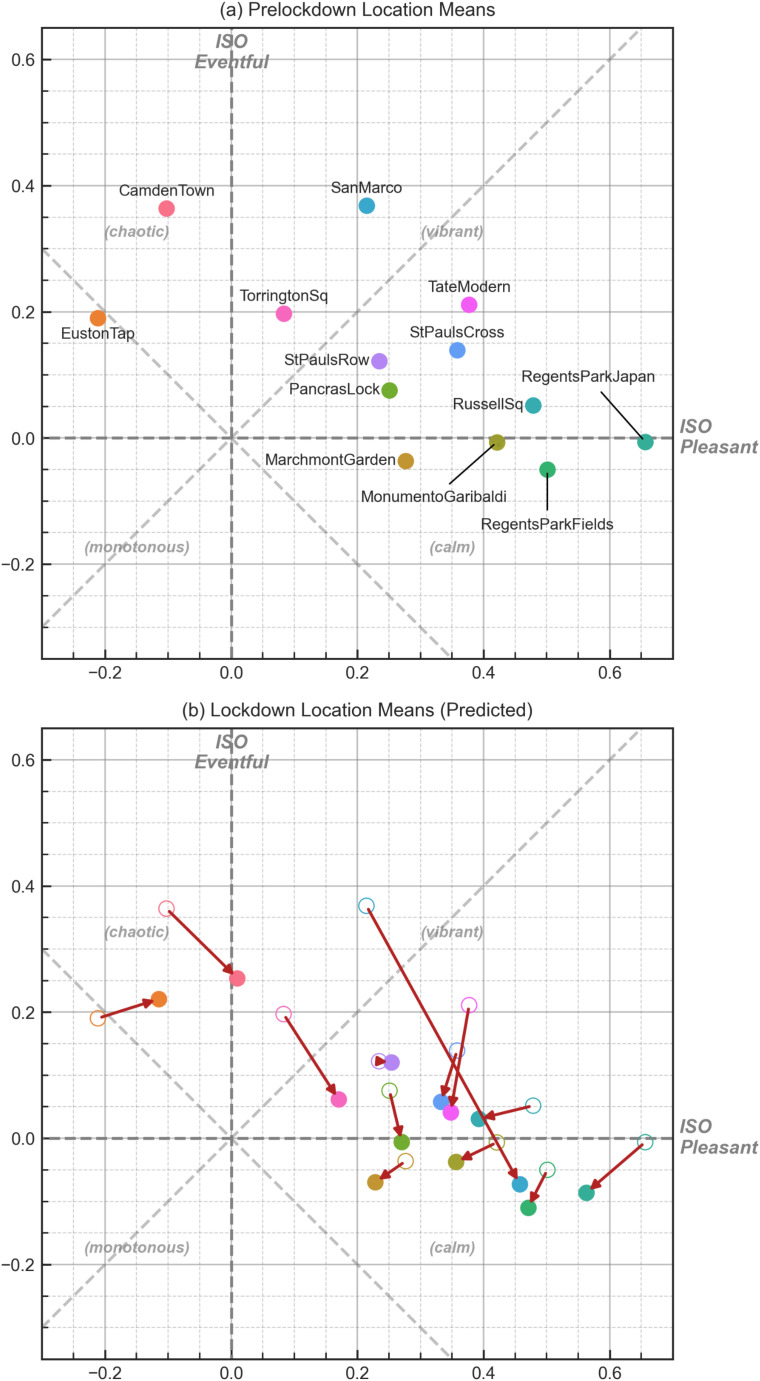
(Color online) The soundscape circumplex coordinates for (a) the mean ISOPleasant and ISOEventful responses for each location and (b) the mean predicted responses based on the recordings made during the lockdown and the change in the location's placement in the circumplex. In (b), the marker outline is shown for the 2019 location, and red arrows indicate the change in the location's coordinates.

Once the two models were built and assessed, they were then applied to the lockdown recording data to predict the new soundscape ISO coordinates. Figure 5(a) shows the pre-lockdown ISO coordinates for each location, and Fig. 5(b) shows how the soundscapes are predicted to have been assessed during the lockdown period. As in the model assessment process, the predicted responses are calculated for each recording individually, and then the mean for each location is calculated and plotted on the circumplex.

In 2019, the majority of the locations in the dataset fall within the “vibrant” quadrant of the circumplex, particularly those which are primarily dominated by human activity (e.g., San Marco, Tate Modern). Camden Town and Euston Tap, which are both, in general, visually and acoustically dominated by traffic, are the only two to be rated as “chaotic,” and no locations are, overall, considered to be “monotonous.” During the 2020 lockdown, there is a general positive move along the “pleasant” dimension and general negative move along the “eventful” dimension, but several different patterns of movement can be noted. These are investigated further in Sec. IV below.

## IV. DISCUSSION

### A. Interpretation of the results

To interpret the results addressing RQ1 and RQ2, it is necessary to separately look into the overall changes in the sound source composition and the affective quality of the soundscapes per location.

#### 1. Change in the sound source composition

The open-ended question about sound sources in the online survey did not reveal a change in the sound source types but rather confirmed that all types were still present in both conditions. The sound source composition question taken from Method A of ISO/TS (2018) revealed a statistically significant reduction in the human sound sources and a significant increase in the perceived dominance of natural sound sources.

The most frequent sound sources detected from the open-ended question correspond to the main four sound source types investigated, which indicated that all types remained present in the lockdown condition (at all of the locations). Although the traffic intensity might have decreased, where the results of the Mann-Whitney *U*-test were inconclusive but supported by the psychoacoustic measurements (Aletta *et al.*, 2020), the traffic-related sound sources were still clearly present.

The sound source composition of an outdoor acoustic environment is extremely complex. Removing one component, such as human sounds, has implications on the whole (Gordo *et al.*, 2021). Testing the effects of this *in situ* is not straightforward, and interpreting this study in line with “what is the impact of human sounds” must be taken within the broader context of the range of conditions, which changed within the acoustic environment. However, looking at the overarching picture, the lockdown condition was a useful and unique case study to understand the impact which human activities—and the human sound source type in particular—can have on soundscape perception of urban open spaces.

#### 2. Predicted relative changes in soundscapes due to COVID-19 restrictions

**FIG. 6.**
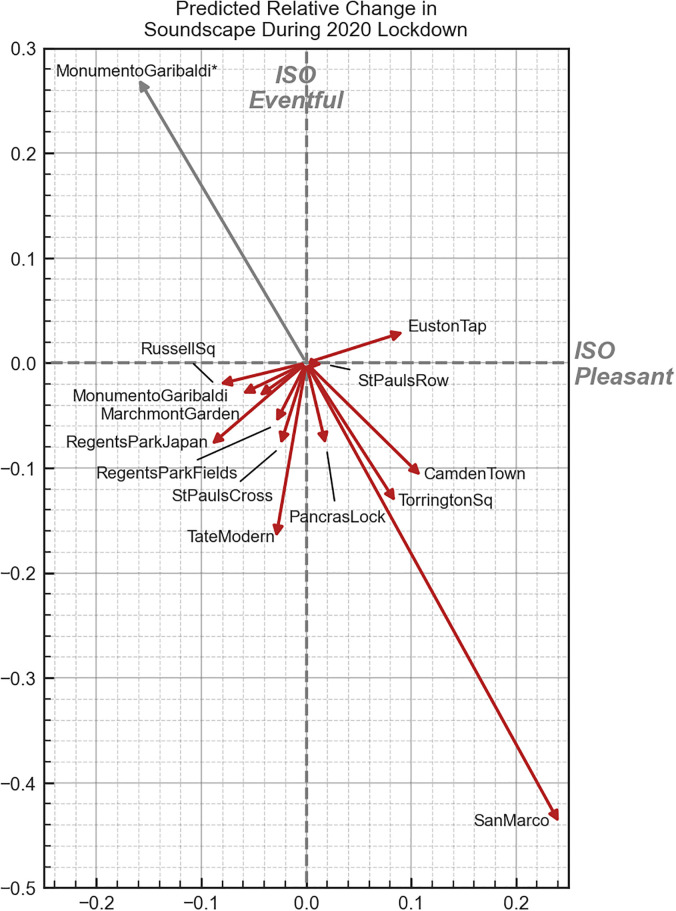
(Color online) The relative change in the soundscape perception in the circumplex due to the COVID-19 lockdowns as predicted by the models, represented as vectors centered on the origin. *The landscaping works dominated session is shown separately as “MonumentoGaribaldi*” with a gray arrow to indicate that this is distinct from the effects of the lockdown changes.

To interpret how the change of the acoustic environment at the locations examined would have been perceived and answer RQ2, the relative change vectors within the circumplex space are shown in Fig. 6. This clearly shows a few different patterns of the soundscape change resulting from the effects of the 2020 lockdown. These can be further looked into depending on the magnitude and direction of change; shifts between the quadrants, shown in Fig. 5; and the sound source composition. The discussion below is organized according to groups of locations, which show similar behaviors in the predicted magnitude and direction of the change, or discusses a single location that is particularly notable.

##### a. Piazza San Marco.

The largest change is seen in Piazza San Marco, with a predicted increase in pleasantness of 0.24 and a decrease in eventfulness of 0.44, enough to move the soundscape out of the vibrant quadrant and into the “calm” quadrant. This extreme change (relative to the rest of the locations) is exactly what would be expected given the unique context of the measurements taken in 2019—the measurement campaign corresponded with Carnevale, a yearly festival which centers around the square. By contrast, because of the particularly strict measures imposed in Italy during the lockdown measurement period, the square was almost entirely devoid of people. What is promising is that without any of this contextual information about the presence or absence of people, our model is able to capture and reflect what may be considered a reasonable and expected direction and scale of change within the soundscape circumplex.

##### b. Locations showing an increase in pleasantness.

The next locations of interest are those which, in the 2019 survey data, were rated as being dominated by traffic noise: Euston Tap, Camden Town, Torrington Square, and Pancras Lock. These are the only locations (besides San Marco) which show a predicted increase in pleasantness. Of these traffic-dominated spaces, the two that were most heavily dominated by traffic noise (Camden Town and Euston Tap) showed the most increase in pleasantness with Torrington Square having slightly less of an increase. Pancras Lock, which was also rated as having high levels of both human and natural sounds, shows only a modest improvement in pleasantness.

##### c. Locations showing a decrease in pleasantness.

Among the locations which are predicted to experience a negative effect on pleasantness, we see a mix of spaces that were assessed as being dominated by human (St. Pauls Cross and Tate Modern) and natural (Regents Park Japan, Regents Park Fields, Russell Square) sounds before the lockdown. It is hard to discern a pattern of difference between these two groups, although it appears that the human-dominated spaces saw a greater reduction in eventfulness compared to the natural-dominated spaces.

In general, we note that most of the spaces experience some degree of reduction in eventfulness. This pattern is mainly consistent with what would be expected from a reduction in the human presence in these spaces (Aletta and Kang, 2018), as reflected by the observation that, for the most part, those spaces which had the most human sounds prior to the lockdown showed the greatest reduction in eventfulness during the lockdown. In particular, Tate Modern, Camden Town, and Torrington Square show the greatest reduction in eventfulness. This appears to be due to these locations showing the greatest reduction in overall *L_A_*_eq_ compared to other locations (8.1, 5.2, and 9.2 dB, respectively) with *L_A_*_eq_ being the most influential feature in the eventfulness model, as shown in Table IV. However, Russell Square also experienced a large decrease in *L_A_*_eq_, on average (10.5 dB), but does not show the same reduction in eventfulness. This appears as a result of the correspondingly large decrease in *S* (1.17 acum), which is not seen at the three previously mentioned locations. Russell Square normally features a medium-sized jet fountain, which was turned off during the lockdowns in 2020 and, therefore, experienced a drop in the overall sound level but an increase in the proportion of low frequency noise to high frequency noise reflected by a decrease in sharpness, which, within the eventfulness model, effectively cancels out the impact of the reduction in *L_A_*_eq_. Whereas the overall sound level has an important impact, to determine the true impact a reduction in sound level may have, it must be taken in context with how the other aspects of the sound will also change.

##### d. Euston Tap.

An unexpected result is that Euston Tap is predicted to experience an increase in eventfulness, and it is unclear whether this accurately reflects the real experience people would have had in the space. Normally, Euston Tap is a mostly outdoor drinking venue located at the entrance to London Euston Station and situated directly along a very busy central London road. During the 2020 survey, the researchers noted that the music and chatter of people from the pub was noticeably missing but that the perceived reduction in road traffic was minimal. Based on the theory of vibrancy, which would suggest it is driven by human presence and sounds (Aletta and Kang, 2018), we would not, therefore, expect a shift in the vibrant direction as indicated here. This discrepancy may reveal a weakness in the context-independent ISOEventful model or it may, in fact, be indicating that at certain thresholds of traffic noise, a reduction in the level—and, hence, a reduction in the energetic masking—will allow other aspects of the sound to influence the perception.

##### e. Monumento Garibaldi.

Finally, special attention should be paid to the results shown for Monumento Garibaldi, which, in 2019, was perceived as a pleasant and slightly calm green space, featuring a gravel walkway. During the first measurement session during the lockdown in 2020, the researcher noted that the soundscape was dominated by landscaping works, in particular, noise from strimmers (or weed whackers). To gain a sample which was more representative of the impact of the lockdowns, the researcher returned another day to repeat the measurements without interference from the landscaping works.

To examine the impact of these two scenarios separately, the prediction model was fitted to the data from the two sessions independently, and the session that was impacted by the landscaping works is shown in Fig. 6 in gray and labeled MonumentoGaribaldi*, whereas the unaffected session is shown in red. In the latter case, the predicted change in soundscape as a result of the lockdown fits neatly into what would be expected and closely matches the predicted behavior of similar locations in London (i.e., Marchmont Garden and Russell Square). On the other hand, the session which was dominated by noise from the strimmers is predicted to have become much more chaotic with a decrease in pleasantness of 0.16 and an increase in eventfulness of 0.27. This indicates that although the model has no contextual information about the type of sound and, in fact, the training data never included sounds from similar equipment, just based on the psychoacoustic features of the sound, it is able to reasonably predict the expected change in the soundscape.

##### f. General notes.

As a whole, the primary impact of the 2020 lockdowns on the soundscapes in London and Venice was an overall decrease in eventfulness. With the exception of Euston Tap, all of the sessions show some degree of reduction in eventfulness, reflecting the general decrease in the sound levels and human sound sources across the locations. The impact of the lockdowns on pleasantness is more mixed and seems to be driven by the previous dominance of traffic noise in the space. However, it could also be noted that although all of the locations experienced a reduction in the sound level, those which are predicted to become more pleasant had an average *L_A_*_eq_ above 60 dB in 2019. By contrast, the locations which were predicted to experience a decrease in pleasantness generally had sound levels below 60 dBA in 2019. This may indicate that reductions in the sound level can improve pleasantness when the sound level exceeds some threshold of around 60–65 dBA but are ineffective when sound levels are below this threshold. Similarly, Yang and Kang (2005) showed that when the sound level is “lower than a certain value, say 70 dB” there is no longer a significant improvement in the evaluation of the acoustic comfort as the sound level reduces. It is unclear at this point where this threshold would lie for pleasantness/annoyance, how strict it may be, or how it is impacted by the sound source composition of the acoustic environment; therefore, further research is needed in this area.

#### 3. Model selection results

The most immediately interesting result of the model building and feature selection process, answering RQ3, is the apparent irrelevance of the location context to the ISOEventful dimension. The MLM structure was chosen because the starting assumption was that the soundscape perception is heavily influenced by contextual factors such as expectations of the space and visual context (Ricciardi *et al.*, 2015). For this modeling, these factors can be considered to be location-level latent variables, at least, partially accounted for by the inclusion of the LocationID as the second-level factor. Whereas this assumption certainly held true for ISOPleasant, our results indicate that these types of contextual factors are not significant for ISOEventful, and do not affect the relationship between the acoustic features of the sound and perception.

In particular, this result may herald a shift in the modeling approach for soundscapes—where previous methods in the soundscape and noise paradigms, have mostly focused on deriving the acoustic models of annoyance (in other words, they have focused on the ISOPleasant dimension), perhaps they should instead consider the acoustic models as primarily describing the eventfulness dimension when considered *in situ*. In addition, this study takes the approach of modeling responses at an individual-level to derive the soundscape assessment of the location. Rather than either attempting to represent the predicted response of an individual person—which is less useful in this sort of practical application—or base the model on average metrics of the location, the goal is instead to characterize the location itself through the aggregated predicted responses of individuals. The authors believe this modeling approach better addresses the practical goal of predictive soundscape modeling and reflects the structure of the data collection.

### B. Limitations of the study

The on-site sampling method was initially not intended as the ultimate characterization of a location's soundscape but rather as a tool for model development. Therefore, the change observed does not necessarily represent the ground truth about the site's soundscape if such a thing exists. Further, the online listening tests took a relatively small but random sample from the available database^1^ and did not include any contextual information. This proved to be sufficient for the purpose of detecting a change in the sound source composition; however, the relatively small sample of recordings included in the online study does limit how representative they are of the location's sound environment as a whole. Similarly, the surveys and recordings taken represent only a snapshot of the soundscape or sound environment for a short period in time. This is a flaw in most soundscape sampling methods presented in the literature and ISO/TS (2018). To truly be said to characterize the soundscape of a space, long-term monitoring and survey methods will need to be developed to capture the changing environmental and contextual conditions in the space. Models of the sort presented here, which are based on measurable quantities, could prove to be useful in this sort of long-term monitoring as they could take continuous inputs from sensors and generate the likely soundscape assessment over time.

The audio-visual interaction forms a key component in people's perception of urban spaces. This consideration has been a strength of soundscape research and incorporated via the use of an *in situ* data collection. However, the visual aspect and, in particular, how the visual environment changed as a result of the lockdown condition, was not considered in this study, reducing the comprehensiveness of the model. This was due primarily to the data collection limitations imposed by the lockdown restrictions, which made it impractical to replicate the 360° videos made during the 2019 sessions. Future work on comprehensive predictive soundscape models should strive to make use of this visual aspect within their considered features.

The limitation of the sound source categorization adopted from the ISO standard is that it may not be clear to a respondent in which category they would place community sounds like church bells and music. This may be particularly relevant for comparing the lockdown condition as, in particular, the ringing of bells for worship varied in different contexts throughout the pandemic. Whether the bells ceased entirely or were increased not only would have an impact on the sound environment, but the purposeful action behind the decision to ring bells may have changed to the public's relationship to and perception of the sound itself (Parker and Spennemann, 2020). The open-ended question on the sound sources, however, revealed the presence of the church bells in both years. Unfortunately, this is a limitation of the sound source categories given by the ISO standard on which this questionnaire was based. A sensible update based on the findings and experiences reported here would be to combine the traffic and other noise categories because separating them does not appear to provide additional information and include a new category, which would in some way encapsulate the types of community sounds for which there is currently not a clear category.

Further, the lockdown condition is likely to cause distortions of the circumplex soundscape perception model. Therefore, it is important to acknowledge that all of the predictions were made for the people with no experience of the pandemic and its psychological effects. Conceptually, this model captured the perceptual mapping (i.e., the relationship between the acoustic indicator inputs and the soundscape descriptor outputs) of people in 2019, but this perceptual mapping is likely to have been affected by the psychological and contextual impacts of the lockdown itself, independent of its changes on the sound environment. Future research might look into potential perception changes in the post-pandemic world.

## V. CONCLUSION

This study demonstrates an application of predictive modeling to the field of soundscape studies. The model building results reveal that within this dataset, an approach based on psychoacoustics can achieve $R^{2}=0.85$ for predicting the pleasantness of locations and $R^{2}=0.715$ for predicting the eventfulness. A modeling focused method of this sort is a key component to the potential scalability of the soundscape approach to applications such as smart city sensing, urban planning, and cost-effective, sustainable design. To demonstrate the usefulness and feasibility of such an approach, we apply our predictive model to a unique case study in which the traditional soundscape survey methods were impossible.

By applying this predictive model to recordings collected during the 2020 lockdown, the change in perception of the urban soundscapes is revealed. In general, the soundscapes became less eventful, and those locations that were previously dominated by traffic noise became more pleasant. By contrast, the previously human- and natural-dominated locations are, in fact, predicted to become less pleasant despite the decrease in the sound levels. Although all sound source categories remained present in both years, overall, in 2020, a decrease in the human sounds' dominance was observed together with an increase in the perceived dominance of natural sounds. Although these results are limited in that they represent one snapshot of the soundscape of the spaces, the success of the model in responding to new and disturbing sound events demonstrates its potential usefulness in long-term monitoring of urban soundscapes.

## Figures and Tables

**FIG. 7.**
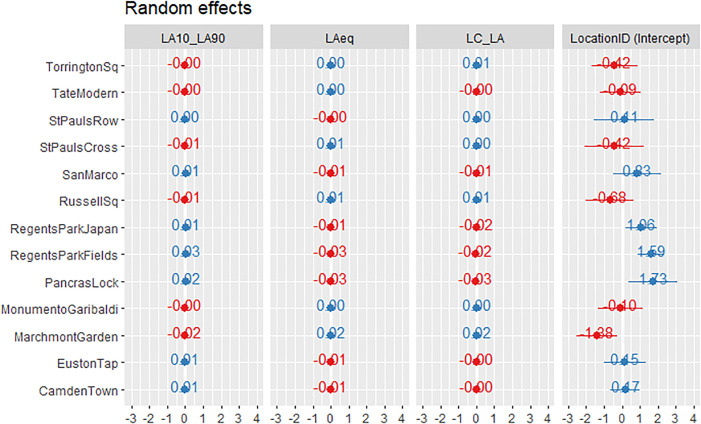
(Color online) The unscaled location-level coefficients for the ISOPleasant model.

## Footnotes

^1^
See https://zenodo.org/record/5654747 for “The International Soundscape Database (V0.2.1)” (Last viewed 11/9/21).

^2^
See supplementary material at https://www.scitation.org/doi/suppl/10.1121/10.0008928 for site descriptions per ISO/TS (2018) featuring the address, overall psychoacoustic characteristics of the location, typical use of each location, and pictures taken during the survey sessions.

^3^
See www.gorilla.sc (Last viewed 11/29/21).

## APPENDIX A: ONLINE QUESTIONNAIRE

For the online questionnaire, as presented to respondents, please see Table V.

**TABLE V.** The questionnaire deployed via the Gorilla Experiment Builder.

Q1	While listening, please note any sound sources you can identify in this sound environment
Q2	To what extent have you heard the following four types of sounds?
	Traffic noise (e.g., cars, buses, trains, airplanes)
	Not at all / A little / Moderately / A lot / Dominates completely
	Other noise **(**e.g., sirens, construction, industry, loading of goods)
	Not at all / A little / Moderately / A lot / Dominates completely
	Sounds from human beings (e.g., conversation, laughter, children at play, footsteps)
	Not at all / A little / Moderately / A lot / Dominates completely
	Natural sounds (e.g., singing birds, flowing water, wind in vegetation)
	Not at all / A little / Moderately / A lot / Dominates completely

## APPENDIX B: MODEL RESULTS

Table VI presents the unscaled coefficients for the ISOPleasant (see Fig. 7) and ISOEventful predictive models. The scaled coefficients are presented in the body of the text to facilitate the comparisons among the various factors. However, we feel it is important to present the unscaled coefficients such that these models could be implemented and compared for future work.

**TABLE VI.** The unscaled linear regression models of ISOPleasant and ISOEventful for 13 locations in London and Venice. Statistically significant *p*-values are highlighted in bold.

	ISOPleasant			ISOEventful		
Predictors	Estimates	CI	*p*	Estimates	CI	*p*
(Intercept)	0.39	0.28–0.50	**<0.001**	−0.77	−1.05–0.48	**<0.001**
*N*_5_	−0.01	−0.01–0.00	**<0.001**			
*S*				−0.17	−0.23–0.12	**<0.001**
FS				−1.36	−2.61–0.11	**0.033**
*T*				0.24	0.08–0.39	**0.002**
*L _A_*_eq_				0.02	0.02– 0.02	**<0.001**
$L_{C\text{eq}}-L_{A\text{eq}}$				−0.01	−0.02–0.00	0.052
Random effects						
$\sigma^{2}$	0.11					
*τ*_00_	$1.01_{\text{LocationID}}$					
*τ*_11_	$0.00_{\text{LocationID}.L_{A\text{eq}}}$					
	$0.00_{\text{LocationID}.L_{A10}-L_{A90}}$					
	$0.00_{\text{LocationID}.L_{C\text{eq}}-L_{A\text{eq}}}$					
ICC	0.90					
*N*	$13_{\text{LocationID}}$					
Observations	914			914		
